# Curiosity-Based Interventions Increase Everyday Functioning Score But Not Serum BDNF Levels in a Cohort of Healthy Older Adults

**DOI:** 10.3389/fragi.2021.700838

**Published:** 2021-08-09

**Authors:** Allison N. Grossberg, Brianne M. Bettcher, Kim A. Gorgens, Aurélie Ledreux

**Affiliations:** ^1^ Knoebel Institute for Healthy Aging, University of Denver, Denver, CO, United States; ^2^ Department of Neurology, University of Colorado Anschutz Medical Campus, Aurora, CO, United States; ^3^ Graduate School of Professional Psychology, University of Denver, Denver, CO, United States

**Keywords:** memory, curiosity, Alzheimer’s disease, amyloid, t-tau, brain-derived neurotrophic factor

## Abstract

An enriched environment is effective in stimulating learning and memory in animal models as well as in humans. Environmental enrichment increases brain-derived neurotrophic factor (BDNF) in aged rats and reduces levels of Alzheimer-related proteins in the blood, including amyloid-β (Aβ) peptides and misfolded toxic forms of tau. To address whether stimulation of curiosity, which is a form of enrichment, may provide a buffer against Alzheimer’s disease (AD), we measured levels of biomarkers associated with AD at baseline and after a 6-week intervention in older adults (>65 years of age) randomized to one of three different intervention conditions. Specifically, in this pilot study, we tested the effectiveness of a traditional, structured learning environment compared to a self-motivated learning environment designed to stimulate curiosity. There were no significant differences from baseline to post-intervention in any of the groups for Aβ42/Aβ40 ratio or t-tau (total-tau) plasma levels. Serum BDNF levels decreased significantly in the control group. Interestingly, individuals who had the lowest serum BDNF levels at baseline experienced significantly higher increases in BDNF over the course of the 6-week intervention compared to individuals with higher serum BDNF levels at baseline. As expected, older individuals had lower MoCA scores. Years of education correlated negatively with Aβ levels, suggesting a protective effect of education on levels of this toxic protein. ECog scores were negatively correlated with BDNF levels, suggesting that better performance on the ECog questionnaire was associated with higher BDNF levels. Collectively, these findings did not suggest that a 6-week cognitive training intervention focused on curiosity resulted in significant alterations in blood biomarkers but showed interesting correlations between cognitive scores and BDNF levels, further supporting the role of this trophic factor in brain health in older adults.

## Introduction

Alzheimer’s disease (AD) is the most common cause of dementia, with an estimated 44 million people living with AD or related dementias worldwide (Nichols et al., 2019). The disease is characterized by synaptic dysfunction, neuronal atrophy of forebrain temporal structures, and protein aggregation. Amyloid precursor protein and tau misfold and aggregate into extracellular Aβ plaques and intracellular neurofibrillary tau tangles (Bloom, 2014). Elevated plasma levels of tau are associated with cognitive impairment and brain atrophy and are predictive of future rates of cognitive decline in AD patients (Gómez-Ramos et al., 2006; Zetterberg et al., 2013; Blennow, 2017; Deters et al., 2017). The two main toxic isoforms of Aβ, Aβ42 and Aβ40 are involved in synaptic loss, cell death, and chronic inflammation (Selkoe, 1999; Selkoe, 2002; Meraz-Ríos et al., 2013). AD is also associated with decreased levels of brain-derived neurotrophic factor (BDNF), a protein that is expressed throughout the brain and promotes the survival, growth, differentiation and regeneration of neurons (Nagahara et al., 2009; Bathina and Das, 2015; Miranda et al., 2019).

During the early stages of their illness, patients with AD exhibit learning and memory deficits and decreased curiosity, a form of intrinsic motivation that fosters active learning, interest, attention, and exploration (Daffner et al., 1992; Daffner et al., 1994; Daffner et al., 2006a; Oudeyer et al., 2016). It is estimated that 65% of patients with AD experience apathy, which is a complex construct that is characterized in part by the loss of goal-directed cognitive activity and spontaneous and/or intrinsically stimulated curiosity (Robert et al., 2009). Apathy is predictive of dementia in patients with mild cognitive impairment (MCI; Lanctôt et al., 2017; Robert et al., 2009; Starkstein, 2006). Thus, apathy and the subsequent loss of exploratory behavior seen in AD may be a prominent feature of the early stages of the disease that can be used in conjunction with other diagnostic criteria and as a potential treatment target.

Some behavioral changes associated with MCI or AD could be related to the progressive downregulation of BDNF that has been observed in patients with AD (Fahnestock et al., 2002). Reduction in BDNF levels may negatively impact synaptic plasticity, neuronal regeneration and growth and may exacerbate deficits in motivation, learning and memory formation (Fischer et al., 1992; Miranda et al., 2019). Additionally, BDNF is known to be critically involved in spatial and contextual learning via its role in hippocampal long-term potentiation (LTP) and synapse formation (Mu et al., 1999; Hall et al., 2000; Vicario-Abejón et al., 2002; Barco et al., 2005; Zagrebelsky and Korte, 2014).

These observations, paired with the current lack of effective pharmacological interventions for AD, have led many researchers to focus their attention on lifestyle interventions that have been shown to increase BDNF levels. Such interventions commonly include various combinations of guided physical activity, nutrition, social interaction and cognitive activity. To the best of our knowledge, there are no cognitive training or rehabilitation studies on the effects of curiosity as a mediator of successful learning and healthy aging and no research groups have investigated whether a curiosity-based intervention might help delay the cognitive decline associated with aging and AD. Research supports the notion that older adults with high levels of curiosity experience increased long-term memory retention (Kang et al., 2009; McGillivray et al., 2015), increased cognitive function and a reduced risk of AD (Daffner et al., 2006b). Curiosity may play a neuroprotective role, similar to global cognitive activity, by activating noradrenergic and dopaminergic systems that modulate learning and memory, reduce inflammation and increase Aβ clearance in the hippocampus and pre-frontal cortex (Feinstein et al., 2002; Heneka et al., 2010; O’Donnell et al., 2012; Torres-Rosas et al., 2014; Yan et al., 2015; Mather and Harley, 2016). Curiosity is also known to be modulated by hippocampus-dependent learning mechanisms (Gruber et al., 2014) and may be well-suited to combat the neuronal atrophy and impaired signaling observed in AD. One possible molecular mechanism for this powerful effect is BDNF signaling in the hippocampus. Animal studies have shown that exposure to complex and novel stimuli promotes neurogenesis and synaptic plasticity, likely through increased production of BDNF (Ickes et al., 2000; Mohammed et al., 2002; Rossi et al., 2006). In a transgenic mouse model of AD, enrichment was found to produce a transient, but significant reduction in levels of tau tangles and Aβ plaques, and an increase in circulating BDNF (Billings et al., 2007). In a recent study, we have shown that cognitive training over a 5-week period was associated with increased serum BDNF levels in older adults, suggesting that cognitive training might be beneficial for brain health with aging (Ledreux et al., 2019). Cognitive training has beneficial effects on mental well-being and cognitive function in healthy older adults as well as in AD (Bamidis et al., 2014; Wirth et al., 2014). The neurobiological mechanisms underlying these effects are still under investigation, although Fissler and collaborators (Fissler et al., 2013) propose that cognitive training might promote neuroplasticity by practice-enhanced strengthening of synaptic structure and function.

To address whether stimulation of curiosity may provide a buffer against the pathological changes seen in AD, we measured blood levels of biomarkers (t-tau, BDNF and Aβ42/Aβ40 ratio) associated with AD pathology in older adults randomized to one of three intervention conditions. Specifically, we tested the effectiveness of a traditional, structured learning environment in which participants attended a rigorous, college-style class, compared to a self-motivated learning environment designed to stimulate curiosity. Curiosity was encouraged via the active design and completion of a project of interest to the study participant. The major objective was to generate a unique set of data on the possible connection between cognitive activity, curiosity, and biomarkers associated with AD pathogenesis. We predicted that participants assigned to one of the intervention groups would exhibit alterations in one or several blood biomarkers as well as improvement in cognitive scores and curiosity levels. We chose to study the effects of our two brief cognitive interventions in an asymptomatic group of older adults to study potential preventive effects on areas of cognition that are vulnerable to AD. Given their importance in AD pathology and neurogenesis, the development of non-pharmaceutical interventions that may lead to decreased levels of Aβ or tau, or increased levels of BDNF are critical.

## Methods

### Participants

Forty-two healthy older adults, ages 65–93, were recruited through flyers and recruitment events hosted by two Denver-based volunteer organizations, the Osher Lifelong Learning Institute (OLLI) and Clermont Park Lifeplan Community. Out of the 42 older adults initially recruited, three subsequently chose not to participate. A total of 39 older adults were randomized into one of three intervention groups to obtain a balanced randomized control trial design. Exclusion criteria for data analysis included participant self-report of cognitive decline, major psychiatric disorders, neurological conditions including neurodegenerative diseases/dementias, epilepsy, or chronic medical conditions such as leukemia, rheumatoid arthritis or chronic infectious conditions including prion disease, HIV, meningitis, and hepatitis. All participants were required to disclose participation in other classes or cognitive training programs during the course of the study. Demographic information for each participant is shown in Table 1. All participants provided written informed consent before participating in the study. The study was approved by the University of Denver Institutional Review Board (IRB).

**TABLE 1:** Participant demographics*.* Numbers are mean values ± SEM for continuous variables and numbers (%) for categorical variables. *p*-values were determined via one-way ANOVA for continuous data and Chi Square tests (>5 cases per contingency table cell) or Fisher’s Exact tests (<5 cases per contingency table cell) for categorical data.

	Total study population	Curiosity group	Traditional group	Control group	*p*-value
Number of participants	39	14	13	12	-
Age (Years)	74.1 ± 1.25	73.8 ± 2.37	76.2 ± 2.28	72.0 ± 1.64	*p* = 0.604
Sex (% Female)	30 (76.9%)	12 (85.7%)	9 (69.2%)	9 (75.0%)	*p* = 0.641
Education (Years)	18.4 ± 0.36	18.6 ± 0.46	17.5 ± 0.78	19.1 ± 0.57	*p* = 0.622
Number of medical conditions	1.38 ± 0.27	1.64 ± 0.50	0.85 ± 0.27	1.67 ± 0.59	*p* = 0.979
Family history of AD (% yes)	13 (33.3%)	3 (21.4%)	4 (30.8%)	6 (50.0%)	*p* = 0.334
Hours of Physical Activity (n = 24)	6.73 ± 0.91	8.38 ± 1.80	4.38 ± 0.99	7.44 ± 1.65	*p* = 0.176
Alcohol Use (% yes)	32 (82.1%)	12 (85.7%)	9 (69.2%)	11 (91.7%)	*p* = 0.342
Depression (% yes)	10 (25.6%)	2 (14.3%)	2 (15.4%)	6 (50.0%)	*p* = 0.077
Sleep Apnea (% yes)	10 (25.6%)	4 (28.6%)	4 (30.8%)	2 (16.7%)	*p* = 0.730
Mild Cognitive Impairment (% yes)	6 (15.4%)	2 (14.3%)	2 (15.4%)	2 (16.7%)	*p* = 1.000

### Experimental Procedure

Prior to beginning their randomly assigned intervention, participants attended one hour-long study visit during which they were provided with information about the study, both verbally and in writing, and were asked to provide written, informed consent. Participants were asked to provide detailed demographic information, including a health history, and were screened for cognitive impairment. The screening procedure included the Montreal Cognitive Assessment (MoCA; Nasreddine et al., 2005) and the Everyday Cognitive Assessment (ECog; Farias et al., 2013). Each participant also filled out a validated curiosity inventory, the Curiosity and Exploration Inventory (CEI-II; Kashdan et al., 2009) to assess their self-reported level of curiosity. After the first study session, the participants were randomized into one of the three study groups, two experimental intervention conditions and a control group in which participants completed their daily activities as normal. The two experimental interventions included a non-structured, project-based class or a structured classroom module. In both intervention groups, the participants attended a class held once per week for 2 hours in the same classroom space for a total of 6 weeks.

### Group 1: Non-traditional “Curiosity” Classroom Intervention

Fourteen participants were randomized into the “non-structured intervention”. The course was designed to be self-motivated, allowing participants to create their own individual project based on an internal desire to learn and explore independently of a structured curriculum. Each student was encouraged to pick a topic, whether academic or personal in nature, which they felt sparked their curiosity. No restrictions were placed on the topic selection or the specific type of project conducted. To ensure full participation in the intervention, participants were asked to track weekly progress on their project using an online time tracking system, Toggl (https://toggl.com). The class was supervised by a facilitator, who was instructed to remain as unbiased as possible so as not to influence the learning experience of the participants. No grades were assigned and no traditional outcome measures of learning (tests, grades, assignments, etc.) were used to assess participants’ progress.

### Group 2: Traditional Classroom Intervention

Thirteen participants were randomized into the “structured intervention group”. The class was modeled after a traditional college level course. This module required participants to complete an assigned writing project on a topic chosen by a qualified facilitator and was designed to increase cognitive activity by requiring participants to attend to and recall new, complex information in a traditional learning context. To ensure the curriculum was broadly interesting and not too academically rigorous for a wide range of participants with differing levels of education, the topic chosen for the class was “Positive Psychology”. The content of the course was developed by the module facilitator, a volunteer with OLLI and an experienced college professor. Participants’ knowledge and progress were assessed using traditional outcome measures of learning such as graded tests and assignments.

### Group 3: Control Group

Twelve participants were randomized into a control group and were asked to complete their daily activities as they normally would. Participants were asked to refrain from participating in a separate class or cognitive training program during the 6-week duration of the study.

### Questionnaires

Participants completed a demographics and medical history form which included standard questions regarding physical activity, cognitive activity, disease history, potential neurological conditions, depression and other relevant neuropsychiatric disorders, current pharmaceutical and recreational drug use, gender, age, diet, level of education, and marital status. To understand physical and cognitive activity, we asked participants to report participation in any form of physical or cognitive activity more than twice per week. The physical activities reported were of mild to moderate intensity and included walking, gardening, yoga, weight training, cardio, aerobics, hiking and golf. Cognitive activities commonly included reading and writing, playing games (brain games, puzzles, board games, etc.) and attending classes. The demographics form was filled out once, prior to the 6-week intervention at the time of consent.

Upon signing the consent form, CEI-II, ECog and MoCA questionnaires were administered. The CEI-II is a curiosity assessment that takes approximately 5–10 min to complete. It has been cross-validated and has shown evidence for construct validity (Kashdan et al., 2009). This scale assesses the mid-range of the latent curiosity trait most reliably. The scores range from one to five with higher scores relating to higher levels of self-reported curiosity. It is a two-factor model that includes five items designed to measure motivation to seek out knowledge and new experiences (Stretching) and five that measure willingness to embrace the novel, uncertain, and unpredictable nature of everyday life (Embracing). The ECog is a self-report questionnaire designed to detect cognitive and functional decline and has been shown to have high internal consistency and excellent discrimination between healthy older adults and those with dementia (Farias et al., 2013). The ECog consists of six subscales including everyday memory, language, visuo spatial and perceptual abilities, planning, organization and divided attention. Participants were asked to rate their ability to perform certain tasks currently as compared to their ability to do the same tasks in the past. The ECog score ranges from one to four with a score of one being high ability and four being low ability. The ECog supplemented the information reported by participants on the MoCA. The MoCA is a 30-item measure of global cognitive function, with scores ranging from 1 to 30. Although the MoCA is not a diagnostic test, scores greater than 25 are considered to be within the normal range while scores ranging from 18 to 25 are suggestive of possible MCI (Milani et al., 2018). Functional domains include visuospatial/executive, naming, attention, language, abstraction, delayed recall and orientation. The MoCA has been validated for a wide range of populations and is commonly used in clinical settings (Rambe and Fitri, 2017).

### Blood Collection and Processing

Blood samples were obtained from 39 individuals. Two tubes of blood were obtained from each participant (approximately 10 ml total) at two time points: at baseline and within 24 h of the final class of the 6-week intervention. A BD Vacutainer SST tube was used for serum collection, containing a clot activator and gel. Serum samples were kept at room temperature for 30 min to allow for clotting. They were then centrifuged at 2,000 × g for 15 min at 4°C. Purple BD Vacutainer tubes, containing EDTA, were used for plasma collection. Plasma samples were kept on ice before centrifugation. They were then centrifuged at 1,500 × g for 15 min at 4°C. Plasma and serum samples were aliquoted into 0.5-ml microtubes and stored at −80°C. All blood collections were conducted between 8 and 11 AM to minimize variation across participants and to account for the impact of natural variations in BDNF levels throughout the day (Giese et al., 2014).

### Serum BDNF Analysis

Levels of serum BDNF were quantified using a sandwich enzyme-linked immunosorbent assay for Human Free BDNF (Human BDNF Quantikine ELISA, R&D Systems, Minneapolis, MN) in accordance with the manufacturer’s instructions and our standard protocol (Bharani et al., 2017, 2020; Håkansson et al., 2017). This assay has an intra-assay and inter-assay variability of 5 and 9% respectively, and a minimum sensitivity of 20 pg/ml. Serum samples were diluted 20-fold in the dilution buffer provided in the assay kit and BDNF concentrations were determined via non-linear regression from the standard curves using GraphPad Prism version 8 (GraphPad Software, San Diego, CA).

### Plasma Levels of Aβ40, Aβ42, and t-tau

Plasma levels of Aβ40, Aβ42, and t-tau were assessed on an SR-X instrument (Quanterix, Lexington, MA) using ultrasensitive single molecule array (Simoa) technology. The commercially available Neurology 3 Plex Advantage Kit (Quanterix, Lexington, MA) was used according to the manufacturer’s instructions. All plasma samples, standards and quality controls were run in duplicates. Plasma samples were thawed on ice and centrifuged at 10,000 × g for 5 min at 4°C prior to analysis.

### Statistical Analysis

Analysis was conducted using data from a total of thirty-nine participants (*n* = 39). First, Shapiro-Wilks tests were performed on average scores from each of the pre- and post-questionnaires (ECog, CEI-II, MoCA) and the raw baseline and post intervention values for each biomarker (Aβ40, Aβ42, t-tau, BDNF) to test the assumption of normality. For analysis of plasma t-tau levels, data from one individual was excluded since the post-intervention time point was a significant outlier compared to all other samples (*n* = 38).

To test for differences between groups in terms of demographic variables (age, gender, years of education, etc.), ANOVA, Chi Square or Fisher’s Exact Tests were used. One-way ANOVA was used for continuous data. Chi Square analyses were used with categorical data that had more than five cases in all cells of the generated contingency tables, and Fisher’s Exact Test was used when there were less than five cases in any one of the cells.

ANCOVA was used to test the differences in the post-intervention levels of each biomarker (Aβ42/Aβ40 ratio, t-tau, BDNF) or clinical outcome measure (ECog, CEI-II, MoCA) of interest between the three intervention groups after controlling for variation in the baseline measures. Exploratory correlational analyses (Pearson’s) were performed to investigate possible relationships between the biomarker and outcome measures and other demographic variables (age, years of education and number of medical conditions).

All statistical analyses and data visualization were performed using RStudio for Mac (RStudio (2021). Boston, MA) or GraphPad Prism (version 8, San Diego, CA). The alpha level for null hypothesis rejection was set at 0.05. Data are presented as mean ± standard error of the mean (SEM), unless otherwise noted.

## Results

### Participant Characteristics

The average participant demographics are shown in Table 1. All participants in the study were between the ages of 65 and 93 with a mean age of 74. There were no significant differences in age between the three groups (F_1,37_ = 0.273, *p* = 0.604). There were more females (*n* = 30) than males (*n* = 9) in the total study population, although the proportion of males and females did not differ significantly between the groups (Fisher’s Exact Test, *p* = 0.641). Participants’ years of education ranged from 12 to 22, with an average of 18. Most participants in the study had either a bachelor’s degree (*n* = 10) or a master’s degree (*n* = 17). Years of education did not differ significantly between groups (F_1,37_ = 0.248, *p* = 0.622).

The participants in this study were generally healthy, active, older adults. The two most commonly reported medical conditions were depression and sleep apnea (Table 1), with a total of 17 individuals reporting one of these two conditions and 3 participants reporting both sleep apnea and depression. In addition, a total of 13 participants reported a family history of AD or dementia and this number was not significantly different between treatment groups (Fisher’s Exact Test, *p* = 0.334). Thirty-four participants reported engaging in regular physical activity (defined as any type of physical activity occurring more than twice per week). Twenty-four participants reported the number of hours they spent engaging in physical activity each week, and the average was 6.7 h, with no significant group difference (F_1,21_ = 1.89, *p* = 0.176). All participants reported regularly participating in cognitively stimulating activities including reading, writing, and playing games (puzzles, word games, Sudoku, etc.).

Six individuals had a score below 25 on the MoCA (see Table 1), which is below the recommended cut-off score for normal cognitive functioning (Milani et al., 2018). Thus, for the purpose of this study, these individuals were defined as having possible MCI. There were no significant differences in the number of participants with possible MCI between the groups (Fisher’s Exact Test, *p* = 1.00).

### Correlation Analyses

Age was negatively correlated with baseline and post-intervention MoCA scores (r = −0.56, *p* < 0.001, *n* = 39 and r = −0.43, *p* = 0.007, n = 37, respectively), suggesting that older individuals had lower MoCA scores compared to younger individuals. We found that Aβ40 and Aβ42 levels at both baseline and post-intervention time points were negatively correlated with years of education (Aβ40: r = −0.34, *p* = 0.035, *n* = 39 and r = −0.39, *p* = 0.015, *n* = 39; Aβ42: r = −0.30, *p* = 0.064, *n* = 39 and r = −0.44, *p* = 0.006, *n* = 39), showing that those with more education had lower Aβ40 and Aβ42 plasma levels. We also observed that, at baseline, the ECog scores were negatively correlated with BDNF levels (r = −0.33, *p* = 0.043, *n* = 39) and with the ratio of Aβ42/Aβ40 (r = −0.38, *p* = 0.018, *n* = 39), suggesting that better performance on the ECog questionnaire was associated with higher BDNF levels and higher Aβ ratio. Further, we found that baseline BDNF levels were positively correlated with the ratio of Aβ42/Aβ40 (r = 0.438, *p* = 0.005, *n* = 39) and negatively correlated with age (r = −0.346, *p* = 0.031, *n* = 39). Overall, these findings suggest a complex relationship between age, cognitive performance, education level, and peripheral levels of BDNF and Aβ.

### Effects of Intervention on AD Biomarkers

Means and standard error of the mean for t-tau, Aβ42/Aβ40 ratio and BDNF levels at baseline and post-intervention are presented for each intervention group in Table 2. At baseline, there was no significant difference between the three intervention groups for t-tau, Aβ42/Aβ40 ratio or BDNF, although there was a trend toward significance for a group difference between baseline ratios of Aβ42/Aβ40 (one-way ANOVA, F_1,36_ = 3.14, *p* = 0.06).

**TABLE 2:** Changes in biomarkers of AD (ratio Aβ42/Aβ40, t-tau and BDNF) in the three study groups*.* Values for t-tau are given in pg/mL and values for BDNF are given in ng/mL. Results are presented as mean ± SEM or percentage (%).

		Curiosity group	Traditional group	Control group
	N =	14	13	12
Aβ42/Aβ40 (*n* = 39)	Baseline	0.024 ± 0.003	0.033 ± 0.003	0.033 ± 0.003
	Post	0.022 ± 0.003	0.030 ± 0.003	0.024 ± 0.004
	Change	−0.002 ± 0.002	−0.002 ± 0.002	−0.009 ± 0.005
	% Change	−8.3%	−6.1%	−27.3%
t-tau (*n* = 38)	Baseline	2.56 ± 0.37	2.40 ± 0.23	2.32 ± 0.15
	Post	2.19 ± 0.21	2.16 ± 0.23	2.06 ± 0.18
	Change	−0.37 ± 0.21	−0.24 ± 0.17	−0.27 ± 0.14
	% Change	−14.3%	−9.9%	−11.6%
BDNF (*n* = 39)	Baseline	24.2 ± 2.08	27.2 ± 2.71	32.1 ± 2.01
	Post	23.4 ± 1.66	27.7 ± 2.00	29.8 ± 2.01
	Change	−0.86 ± 1.05	0.52 ± 1.55	−2.27 ± 0.77
	% Change	−3.6%	1.9%	−7.1%

**FIGURE 1:**
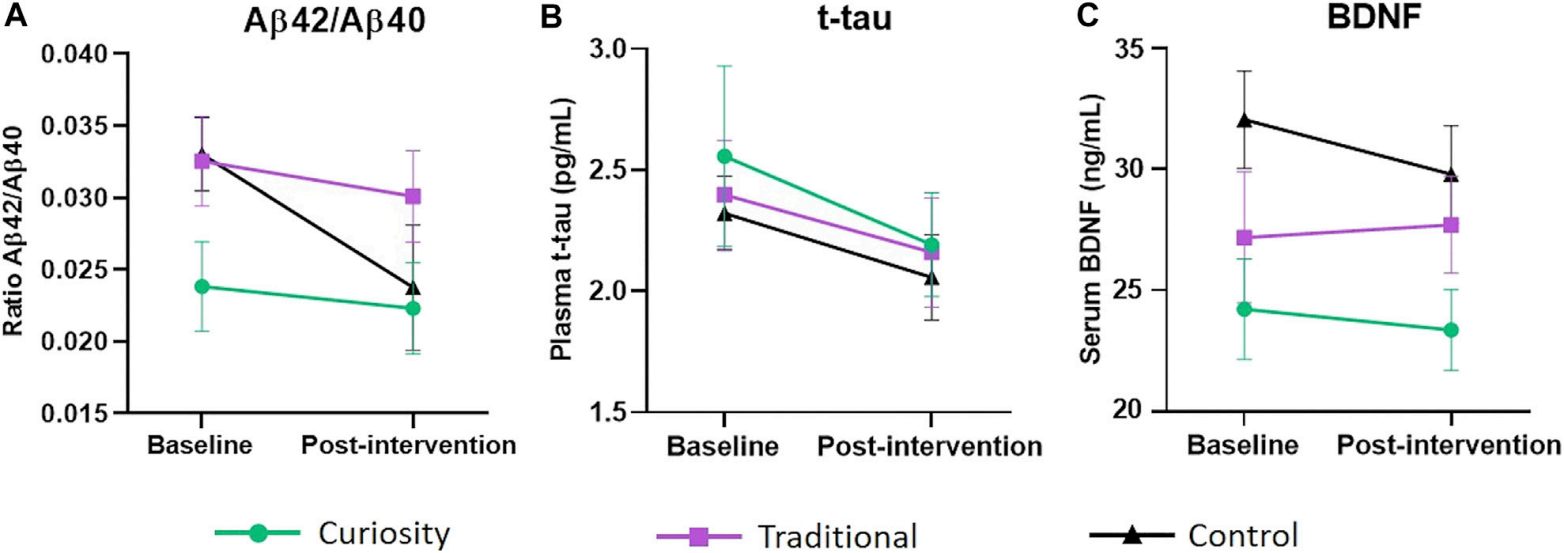
Change in Aβ42/Aβ40 ratio, t-tau and BDNF levels from baseline to post-intervention. **(A)** Change in plasma Aβ42/Aβ40 ratio from baseline to post-intervention in the curiosity group (shown in green), traditional group (shown in purple) and control group (shown in black). Although the control group exhibited a steeper decline, an ANCOVA did not reveal any significant group difference. **(B)** Change in plasma t-tau levels (pg/ml) from baseline to post-intervention in the curiosity group, traditional group and control group. No significant group difference was found. **(C)** Change in serum BDNF levels (ng/ml) from baseline to post-intervention in the curiosity group, traditional group and control group. BDNF levels decreased significantly in the control group (*p* = 0.01). However, there were no significant differences in BDNF concentration between the three intervention groups. All data were analyzed using ANCOVA with post-hoc Bonferroni adjustment. Data are represent as mean ± SEM.

Over the course of the 6-week intervention, the Aβ42/Aβ40 ratio decreased in all three groups, with the largest decrease associated with the control group (Paired *t*-test, *p* = 0.07) (see Table 2). However, an ANCOVA did not reveal any significant group difference (Figure 1A). Levels of t-tau decreased in a similar way in the three groups and there was no significant group difference (Figure 1B). Post-intervention BDNF levels were significantly lower than baseline levels in the control group (Paired *t*-test, *p* = 0.01) while no significant change was observed in the traditional and curiosity groups (Table 2). However, an ANCOVA revealed no significant group difference (Figure 1C).

To understand the potential effects of possible MCI (defined by a MoCA score lower than 25) on biomarkers for AD, we compared participants with MCI (*n* = 6) to those who were cognitively within normal range (*n* = 33) using independent samples *t*-tests. There were no significant differences in baseline or post-intervention levels of BDNF, Aβ42/Aβ40 or t-tau between the two groups. In addition, average scores on the CEI-II and ECog did not differ between participants with MCI and those without. These findings were most likely due to a small sample size for the possible MCI cases versus the controls.

### Effect of Baseline BDNF Levels on BDNF Changes

**FIGURE 2:**
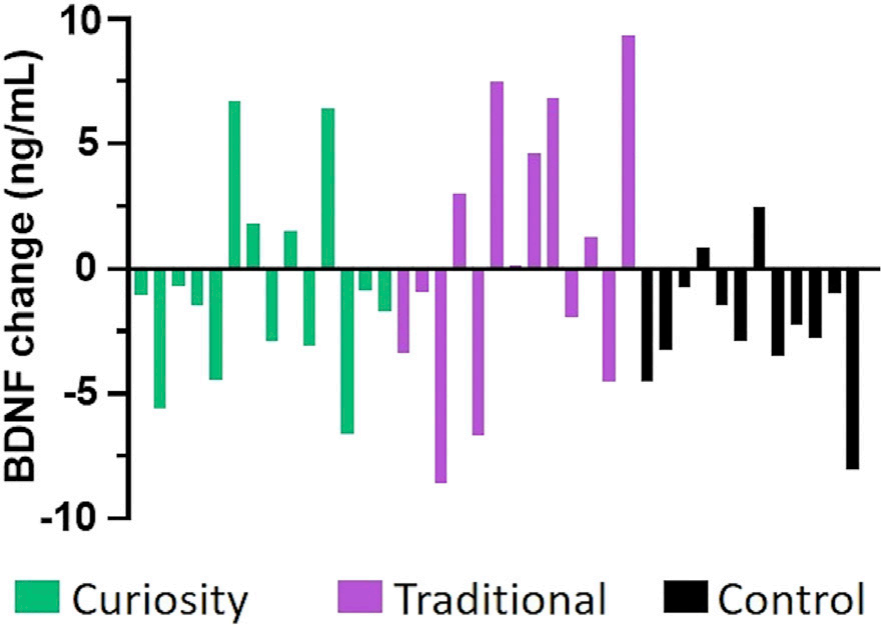
Change in serum BDNF levels from baseline to post-intervention. Each bar depicts the change in BDNF level (in ng/mL) between the post-intervention value and the baseline value for each participant in the curiosity group (shown in green), the traditional group (shown in purple), and the control group (shown in black). A higher proportion of participants in the two intervention groups experienced an increased in BDNF levels, suggesting that both interventions resulted in a positive change in BDNF levels.

Although there were no significant differences between the groups for BDNF using an ANCOVA, we noticed individual differences in BDNF response magnitude across the three intervention groups (Figure 2). Eleven individuals (41%) in the traditional and curiosity groups experienced an increase (ranging from 0.13 to 9.35 ng/ml) in BDNF compared to only two in the control group (17%). This result may indicate that study participants who received either cognitive intervention were more likely than those in the control group to experience an increase in serum BDNF levels.

**FIGURE 3:**
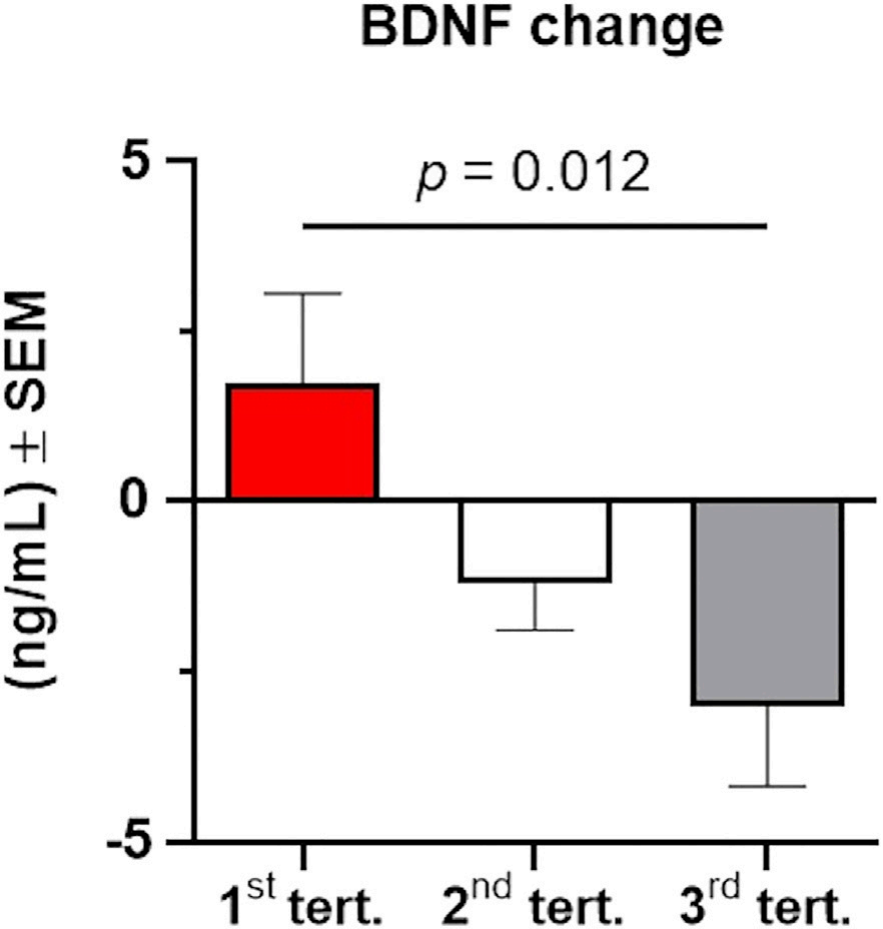
Changes in serum BDNF levels from baseline to post-intervention depend on baseline levels of BDNF. The total cohort of participants was stratified in three groups according to their baseline BDNF levels using tertiles. BDNF change was significantly higher (*p* = 0.012) in individuals with lower BDNF at baseline (1st tertile, shown in red) compared to individuals with higher levels of BDNF at baseline (2nd tertile, shown in grey; 3rd tertile, shown in grey). Data were analyzed using a one-way ANOVA with post-hoc Bonferroni adjustment. Error bars represent the SEM.

We next examined the effects of baseline BDNF levels on post-intervention BDNF changes to investigate if BDNF responses would be more restricted in individuals with higher baseline BDNF levels, thus suggesting a ceiling effect. We found a negative correlation between baseline BDNF levels and BDNF level changes (*r* = 0.572, *p* < 0.001, *n* = 39), suggesting that lower BDNF levels at baseline were associated with higher BDNF level changes. Given this finding, we investigated whether there might be a ceiling effect for BDNF, such that participants with a higher baseline BDNF displayed a smaller BDNF increase after the 6-week intervention. Considering the entire cohort, we divided baseline BDNF values into tertiles, and then compared the change in BDNF between the two time points in the three tertile groups using a one-way ANOVA. We found a significant difference between the three tertile groups (F_2,36_ = 4.844, *p* = 0.014, partial η^2^ = 0.212), with participants with the lowest BDNF levels at baseline experiencing a significantly higher (Bonferroni multiple comparison test: adjusted *p* = 0.012) increase of BDNF levels over the 6-week intervention (1st tertile; 1.73 ± 1.32) compared to those in the 3rd tertile who experienced a decrease in BDNF levels (3rd tertile: −3.02 ± 1.16; Figure 3). Although preliminary, this suggests that cognitive training may have the most substantial impact on individuals who have low levels of BDNF prior to intervention compared to those with higher baseline levels of BDNF.

### Effects of Intervention on Memory, Curiosity and Everyday Cognition Tasks

Before statistical analyses of performance change in the cognitive and curiosity batteries were conducted, a Cronbach’s alpha analysis was used to assess the internal consistency of the questionnaires administered. All three questionnaires (CEI-II, MoCA and ECog) were found to be reliable (α = 0.84; α = 0.57, α = 0.86, respectively).

Average CEI-II, ECog and MoCA scores changed only moderately over the course of the 6-week intervention (Table 3). Nonetheless, some interesting trends could be observed. For example, CEI-II scores increased only in the curiosity group, although the difference did not reach significance (paired *t* test: *p* = 0.08), likely because of the small number of participants. ECog scores decreased significantly in the curiosity and traditional groups (paired *t* test: *p* = 0.018 and *p* = 0.048, respectively), suggesting better performance on this test in these intervention groups while the control group saw no significant change. ANCOVA with baseline scores as covariate showed a trend toward significance for ECog scores (F_1,34_ = 3.08, *p* = 0.06). No significant group differences were found for MoCA or CEI-II scores. We did not find any effects of gender, education level or family history of AD/dementia on average questionnaire scores in any of the groups.

**TABLE 3:** Changes in questionnaire scores (CEI-II, MoCA, ECog) from baseline to post intervention in the three study groups*.* Values for CEI-II and ECog are average scores and values for MoCA are total scores. Results are presented as mean ± SEM or percentage (%).

		Curiosity group	Traditional group	Control group
	N =	14	13	12
CEI-II (n = 39)	Baseline	3.56 ± 0.14	3.27 ± 0.23	3.69 ± 0.22
	Post	3.82 ± 0.16	3.16 ± 0.31	3.67 ± 0.22
	Change	0.26 ± 0.14	−0.11 ± 0.37	−0.03 ± 0.18
	% Change	7.3%	−3.4%	−0.8%
MoCA (n = 39)	Baseline	26.4 ± 0.88	27.5 ± 0.57	26.3 ± 0.69
	Post	25.9 ± 1.16	27.0 ± 0.37	26.5 ± 0.59
	Change	−0.57 ± 0.69	−0.33 ± 0.56	0.09 ± 0.67
	% Change	−2.2%	−1.2%	0.3%
ECog (n = 39)	Baseline	1.55 ± 0.12	1.54 ± 0.08	1.48 ± 0.12
	Post	1.31 ± 0.05	1.42 ± 0.08	1.45 ± 0.13
	Change	−0.24 ± 0.09	−0.12 ± 0.05	−0.00 ± 0.05
	% Change	−15.5%	−7.8%	−0.4%

## Discussion

In this novel pilot study, older adults (ages 65–93) who were asymptomatic to AD were randomized into one of three different brief intervention conditions including a traditional, structured learning environment, a self-motivated learning environment designed to stimulate curiosity, and a non-active control group. The goals of the study were to investigate the effectiveness of these different cognitive interventions on curiosity levels, biomarkers of AD and measures of cognitive function over a 6-week period and to examine individual differences that may alter responses to cognitive intervention and differentially affect changes in Aβ42/Aβ40 ratio, BDNF, and t-tau blood levels over time.

Our group has previously reported a significant increase in serum BDNF levels after a 5-week long computer-based cognitive intervention in healthy older adults that involved 35 min cognitive training sessions 5 days a week (Ledreux et al., 2019). Based on these findings, we had hypothesized that both the self-motivated and traditional learning experiences would result in significant BDNF increases. We found that BDNF levels decreased significantly in the Control group while remaining stable in the traditional and curiosity groups. A higher proportion of participants experienced increased BDNF levels in the two intervention groups compared to the control group, although this difference was not statistically significant. In our previous study (Ledreux et al., 2019), each intervention group included at least 29 participants, which could explain differences in significance between the two studies. Moreover, it is very likely that the frequency and duration of the intervention were not long enough to produce significant changes in BDNF levels, or that the study was not powered enough to reveal statistical significance. Other studies examining the effects of cognitive training on BDNF levels usually have participants engage in the intervention several times per week (Farina et al., 2006; Buschert et al., 2010; Buschert et al., 2011; Kim et al., 2016). In addition, there is evidence that BDNF is required for initial long-term potentiation (LTP) in the brain but must be sustained at high levels to maintain LTP (Aicardi et al., 2004). Thus, BDNF levels might have been elevated in the short-term, i.e., just after each cognitive intervention, but may have not been sustained long enough or at high enough levels to induce detectable changes or improvements in cognition and memory performance. Interventions during which participants attend classes several times per week or over a longer time period may lead to a more robust stimulation of BDNF signaling, neurogenesis, and protective effects in brain regions associated with learning and memory. This will be the focus of future studies.

Similar to our previous findings (Ledreux et al., 2019), data from the current cohort suggest a potential ceiling effect for BDNF, such that participants can be classified as “low responders” or “high responders”, depending on whether they exhibited higher or lower baseline BDNF levels, respectively. Interestingly, correlation analyses in the current cohort revealed that BDNF levels were affected by age, with lower baseline levels seen in the oldest participants. There was also a negative correlation between BDNF levels and ECog scores, potentially suggesting that successful performance in this task demands appropriate BDNF levels. Our findings herein are corroborated by previous work, showing for example a positive correlation between serum BDNF levels and performance on attention and executive domains of cognitive batteries (Costa et al., 2015). Lommatzsch et al. (2005) showed a significant negative correlation between age and BDNF levels in a cohort of healthy adults (20–60 years of age). Similar results have been obtained for individuals over 65 years of age as well (Mizoguchi et al., 2020). These studies collectively suggest that age-related memory function is heavily associated with reduction in BDNF levels. Our study confirms this idea.

Curiosity levels did not change significantly in any of the three intervention groups following the 6-week intervention. Interestingly, however, curiosity levels only increased in the curiosity group compared to the traditional and control groups. Designing an intervention that stimulates curiosity in a diverse group of individuals is challenging given the lack of a widely accepted definition of curiosity (Berlyne, 1954; Loewenstein 1994; Silvia, 2005; Litman 2008; Silvia, 2008; Kidd and Hayden, 2015; Oudeyer et al., 2016). Thus, it is possible that the intervention did not effectively stimulate curiosity enough to observe statistically significant differences, especially given the short duration (6 weeks) and the infrequent timing of the classes (once per week). The curiosity-based course was intended to provide participants with the opportunity to seek out information out of an intrinsic desire to learn, which is in line with the contemporary view of curiosity (Kidd and Hayden, 2015). In contrast, the curriculum for the traditional course was designed to do just the opposite—to motivate participants extrinsically using outcome measures of learning (i.e., grades). Although it is challenging to directly measure whether a study participant is motivated intrinsically or extrinsically, our finding that curiosity only increased in the curiosity group may provide some support to the idea that curiosity is an intrinsically motivated information-seeking drive.

It is possible that the cognitive intervention succeeded in increasing curiosity levels in the curiosity group but failed to elicit meaningful changes in AD biomarkers because curiosity does not provide the same beneficial effects as more structured or rigorous learning or is not substantially different from structured learning. It is also possible that curiosity alone may not have the same impact as more focused learning that requires memory and recall of new information, which may promote LTP in the brain. Rather, curiosity may serve to enhance structured learning by keeping individuals interested and motivated. The possible synergistic relationship between curiosity and structured learning interventions should be investigated in future work. Another possible explanation for the lack of change in AD biomarkers, is that the duration and the frequency of the intervention were not high enough to elicit any detectable changes in Aβ42/Aβ40 ratio, BDNF, or t-tau as a result of increased curiosity levels. Importantly, the curiosity levels of all participants enrolled in the study were high at baseline—most likely due to the population characteristics of the cohort. In the future, an effort should be made to recruit study participants who are not typically engaged in community-based classes or cognitive training programs and who may represent a greater spectrum on a continuum of curiosity levels.

While ratios of Aβ42 to Aβ40 decreased in all groups, we found that the Aβ42/Aβ40 ratio decreased more substantially in the control group. In a study investigating the effects of cognitive-motor dual-task training in healthy elderly people, Yokoyama et al. (2015) found that 12 weeks of intervention reduced plasma Aβ ratio, but the authors did not provide an explanation for this result. Giudici et al. (2020) demonstrated that low plasma Aβ42/Aβ40 ratio was associated with more pronounced cognitive decline over time (Giudici et al., 2020). Moreover, in a recent study, Doecke et al. (2020) demonstrated that plasma Aβ ratios were predictive of amyloid deposition in the brain as measured by PET imaging (Doecke et al., 2020). While unlikely over the short period of time used in this study (6 weeks), the decreased plasma Aβ ratio in the control group could be suggestive of an increased risk for cognitive decline. A larger sample size and longer intervention times are needed to better understand whether plasma levels of Aβ42 and Aβ40 are good markers for prediction of various stages of cognitive decline with aging.

Baseline and post-intervention levels of Aβ40 and Aβ42 were negatively correlated with level of education such that high Aβ levels were related to less years spent in school. This has been previously reported in a study by Yaffe et al. (2011) who found that individuals with less education had higher plasma Aβ levels. Scores on the MoCA were largely unchanged in all three intervention groups after the 6-week study, which is expected. We did find a negative correlation between BDNF levels and ECog scores, suggesting that individuals who performed better on everyday cognitive tasks had higher BDNF levels. Interestingly, we did find that ECog scores decreased significantly in the curiosity and traditional groups compared to the control group, which suggests that cognitive intervention did have a positive impact on participants everyday functioning. We also found that age was negatively correlated with baseline- and post-intervention MoCA scores which suggests that the best predictor of cognitive decline is aging, as older individuals are more likely to have lower MoCA scores. This is not surprising since it is well-known that age is the number one risk factor for AD (Armstrong, 2019). In addition, we found that BDNF levels were positively correlated with the ratio of Aβ42 to Aβ40. This replicates and contributes to other studies that have shown that higher BDNF levels and increased Aβ ratio are indicative of a lower risk for dementia (van Oijen et al., 2006; Weinstein et al., 2014; Chouraki et al., 2015; Lopez et al., 2019; Miranda et al., 2019). These results are important since characterization of the very early clinical presentation of AD is difficult given the mild and varied nature of cognitive symptoms and the difficulty in discriminating between early AD and normal aging (Knopman et al., 2000; Schneider et al., 2009). Researchers across disciplines believe that the underlying pathology of AD begins many years, or even decades, before the clinical manifestation of the disease (Caselli and Reiman, 2013). Thus, understanding how BDNF, Aβ ratio, ECog and MoCA are related is critical for their continued use as diagnostic biomarkers for early detection of MCI and AD.

### Study Limitations

There are several limitations in the present study. A larger sample size is needed to evaluate effects of cognitive intervention on AD biomarker levels over time while controlling for subject variability and differences in gender, age and other characteristics (Lommatzsch et al., 2005). We chose to enroll healthy older adults without AD given the preliminary nature of the study. Future studies should be focused on investigating the effects of different cognitive interventions in older adults with MCI and AD in order to better understand the impact of curiosity training on progression of the disease. This study utilized sampling of convenience to recruit participants. Thus, the sample reflects a population of highly educated, white adults, which is another limiting aspect of this study. Future studies should include demographically diverse populations. Finally, participants attended classes once per week for 2 h over a 6-week period which may not have been enough engagement to elicit meaningful changes in AD biomarkers, cognitive function, or curiosity levels. In the future, we hope to examine the impact of a similar intervention at an increased frequency and over a longer duration.

## Conclusion

The present study is the first to describe the impacts of a curiosity based cognitive intervention for healthy older adults compared to a more traditional, structured intervention. We found that serum BDNF levels stayed stable over the course of 6-weeks in individuals randomized into the traditional group compared to the curiosity group and the sedentary control group, although this result was not statistically significant. Interestingly, we also found that participants with the lowest BDNF levels at baseline experienced a greater increase in BDNF levels over the course of the intervention. Additionally, we found that ECog scores improved significantly in the traditional and curiosity groups compared to the control group and ECog scores were negatively correlated with BDNF levels. These results suggest that our interventions resulted in better performance on the ECog questionnaire which was associated with higher BDNF levels. In summary, our findings suggest that cognitive activity may increase everyday functioning and BDNF levels, at least in a subset of vulnerable participants who began the 6-week intervention with low BDNF levels, which may help reduce the risk for cognitive decline and AD.

## Data Availability

The raw data supporting the conclusions of this article will be made available by the authors, without undue reservation.
